# Glucocorticoid reduction with baricitinib in rheumatoid arthritis refractory to prior therapies: a 24-week real-world analysis

**DOI:** 10.3389/fmed.2026.1797242

**Published:** 2026-03-19

**Authors:** Takashi Yamane, Ayaka Inoue, Noriaki Yasuda, Takahisa Ohnishi, Akira Hashiramoto

**Affiliations:** 1 Department of Rheumatology, Kakogawa Central City Hospital, Kakogawa, Japan; 2 Department of Biophysics, Kobe University Graduate School of Health Sciences, Kobe, Japan

**Keywords:** baricitinib, difficult-to-treat rheumatoid arthritis, glucocorticoid tapering, Janus kinase inhibitor, real-world study, rheumatoid arthritis

## Abstract

**Introduction:**

In a real-world cohort of rheumatoid arthritis (RA) patients who were unable to discontinue glucocorticoids (GCs) despite prior therapies, we aimed to evaluate the GC-sparing effect of baricitinib (BARI) and to identify clinical factors associated with GC reduction.

**Materials and methods:**

This single-center retrospective observational study included RA patients who initiated BARI while receiving systemic GCs between 2018 and 2024. Disease activity and daily GC dose were assessed at baseline and at weeks 4, 12, and 24. The primary outcome was the percent reduction in daily GC dose at week 24. Linear and logistic regression analyses were performed to identify factors associated with GC reduction and discontinuation, with two-variable multivariable models constructed to avoid overfitting.

**Results:**

Among 177 patients who initiated BARI, 39 (22%) were receiving GCs at treatment initiation and were included in the analysis. After prior exposure to biologic or targeted synthetic DMARDs in 31 patients (79%), 34 (87%) had moderate or higher disease activity by the Clinical Disease Activity Index at baseline, and 27 (69%) had comorbid interstitial lung disease. The median daily GC dose decreased from 3.0 mg/day at baseline to 1.0 mg/day at week 24 (*P* < 0.001), with a significant reduction observed as early as week 4. The median GC reduction rate at week 24 was 66%, with 18 of 39 patients (46%) achieving a ≥ 50% reduction and 10 patients (26%) discontinuing GCs entirely. Disease activity improved significantly, with 62% of patients achieving remission or low disease activity by week 24. In linear regression analysis, refractoriness to ≥ 2 prior biologic or targeted synthetic DMARDs was independently associated with a smaller degree of GC reduction. No unexpected safety signals were observed.

**Conclusion:**

In this real-world cohort of GC-dependent RA patients with persistent disease activity and frequent ILD comorbidity despite prior therapies, BARI enabled clinically meaningful GC reduction and improved disease activity within 24 weeks. Resistance to multiple prior b/tsDMARDs emerged as a key barrier to GC tapering, highlighting the importance of introducing JAK inhibitors before progression to a difficult-to-treat state.

## Introduction

In the current treatment algorithm for rheumatoid arthritis (RA), glucocorticoids (GCs) are recommended only as short-term adjunctive therapy at the initiation of methotrexate or during switching of conventional synthetic disease-modifying antirheumatic drugs (csDMARDs) (1). Although even low-dose GC use has been associated with increased risks of infection, osteoporosis, and cardiovascular events (2–4), and recent treatment guidelines for autoimmune diseases, including systemic lupus erythematosus and connective tissue disease–associated interstitial lung disease (CTD-ILD), emphasize minimizing or discontinuing GC use whenever possible (5, 6).

In real-world practice, however, tapering or discontinuing GCs remains particularly challenging in patients with RA complicated by comorbid ILD or CTD, such as Sjögren’s disease or positive antinuclear antibody (ANA) status, where disease activity often persists despite optimized csDMARD or biologic therapy (7, 8). Given that concurrent use of biologic or targeted synthetic DMARDs (b/tsDMARDs) and GCs further increases infection risk, reducing GC exposure remains a crucial therapeutic goal (9).

Recently, the concept of difficult-to-treat rheumatoid arthritis (D2T-RA) has gained attention. According to the EULAR definition, D2T-RA is characterized by failure to achieve disease control despite treatment with at least two b/tsDMARDs with different mechanisms of action (10). D2T-RA patients often have persistent inflammation, progressive joint damage, and are frequently unable to discontinue GCs (11). Long-term observational data have shown that prolonged GC use and delayed initiation of methotrexate within three months of disease onset are predictors of progression to D2T-RA (12). Therefore, optimal introduction of therapies and early GC tapering are essential to prevent D2T disease evolution.

Unlike bDMARDs that target a single extracellular cytokine or receptor, Janus kinase (JAK) inhibitors modulate intracellular signaling pathways shared by multiple pro-inflammatory cytokines through the JAK–STAT signaling cascade (13). By simultaneously attenuating several inflammatory cascades, JAK inhibition may achieve broader suppression of immune activation, thereby potentially facilitating earlier and more stable GC tapering in patients with persistent disease activity.

Baricitinib (BARI), a selective JAK 1/2 inhibitor, has demonstrated strong efficacy in RA and other autoimmune diseases (14). Its safety profile is comparable to that of TNF inhibitors (15). Recent studies have suggested that JAK inhibitors, including BARI, exert potent GC-sparing effects across multiple patient populations. Although the effect may be attenuated in D2T-RA, JAK inhibitor (JAKi) still demonstrates clinically meaningful benefit in this population (16). However, real-world evidence on the GC-sparing effect of BARI and the factors associated with successful GC tapering remains limited.

This study aimed to evaluate the GC-sparing effect of BARI over 24 weeks and to identify baseline factors associated with achieving substantial GC reduction in RA patients receiving concomitant GCs.

## Materials and methods

This retrospective observational study was conducted in the Department of Rheumatology, Kakogawa Central City Hospital, Japan. Patients who initiated BARI between January 2018 and July 2024 were identified from electronic medical records. Among them, those receiving systemic GC therapy at BARI initiation were included in the analysis. Baseline clinical characteristics included age, sex, disease duration, seropositivity for rheumatoid factor (RF) and/or anti-citrullinated protein antibody (ACPA), Clinical Disease Activity Index (CDAI) scores, initial BARI dose (2 or 4 mg), concomitant csDMARDs, prior exposure to b/tsDMARDs, presence of ILD or CTD, and daily GC dose (prednisolone equivalent). CDAI and daily GC dose were evaluated at baseline, week 4, week 12, and week 24 after BARI initiation. In routine clinical practice, BARI was prescribed at 2 or 4 mg at the physician’s discretion. The 2 mg dose was generally selected for patients with renal impairment, frailty, or financial reasons, whereas 4 mg was used for typical candidates. Because both doses are commonly interchanged or adjusted in real-world care, analyses were performed regardless of the initial dose. GC tapering followed a treat-to-target approach aligned with current recommendations to reduce GCs as rapidly as clinically feasible (1). Dose reduction was initiated after improvement in disease activity and continued as long as no clinical worsening occurred. The pace and magnitude of tapering were individualized at the treating physician’s discretion, taking into account disease stability and patient comorbidities.

In addition, BARI dose (2 or 4 mg) was included as an independent variable in the regression models to adjust for its potential influence.

The primary outcome was the percent reduction in daily GC dose at week 24 compared with baseline. Secondary outcomes included changes in CDAI and the proportions of patients achieving CDAI remission or low disease activity (LDA) during the 24-week period.

Continuous variables were expressed as mean ± standard deviation (SD) or median (interquartile range [IQR]), and categorical variables as counts and percentages. Changes in CDAI and GC dose over time were analyzed using the Friedman test, followed by *post hoc* pairwise comparisons with Bonferroni correction. Differences in categorical outcomes (CDAI remission/LDA and GC reduction categories) were analyzed using Cochran’s Q test and McNemar’s test with Bonferroni adjustment. Factors associated with GC reduction were assessed using linear regression analyses, with clinically relevant variables entered as independent factors. In addition, logistic regression analysis was performed to evaluate factors associated with achieving GC-free status at week 24. Given the limited sample size, two-variable multivariable models were constructed to avoid overfitting. All analyses were performed using SPSS version 26 (IBM Corp., Armonk, NY, USA), and *P* < 0.05 was considered statistically significant. Analyses were conducted using an on-treatment approach. Patients who discontinued BARI were included up to the time of discontinuation, and analyses at each time point were based on available data. This study was approved by the Institutional Review Board of Kakogawa Central City Hospital (approval number: #2025-37) and conducted in accordance with the Declaration of Helsinki. The requirement for informed consent was waived due to the retrospective nature of the study.

## Results

**FIGURE 1:**
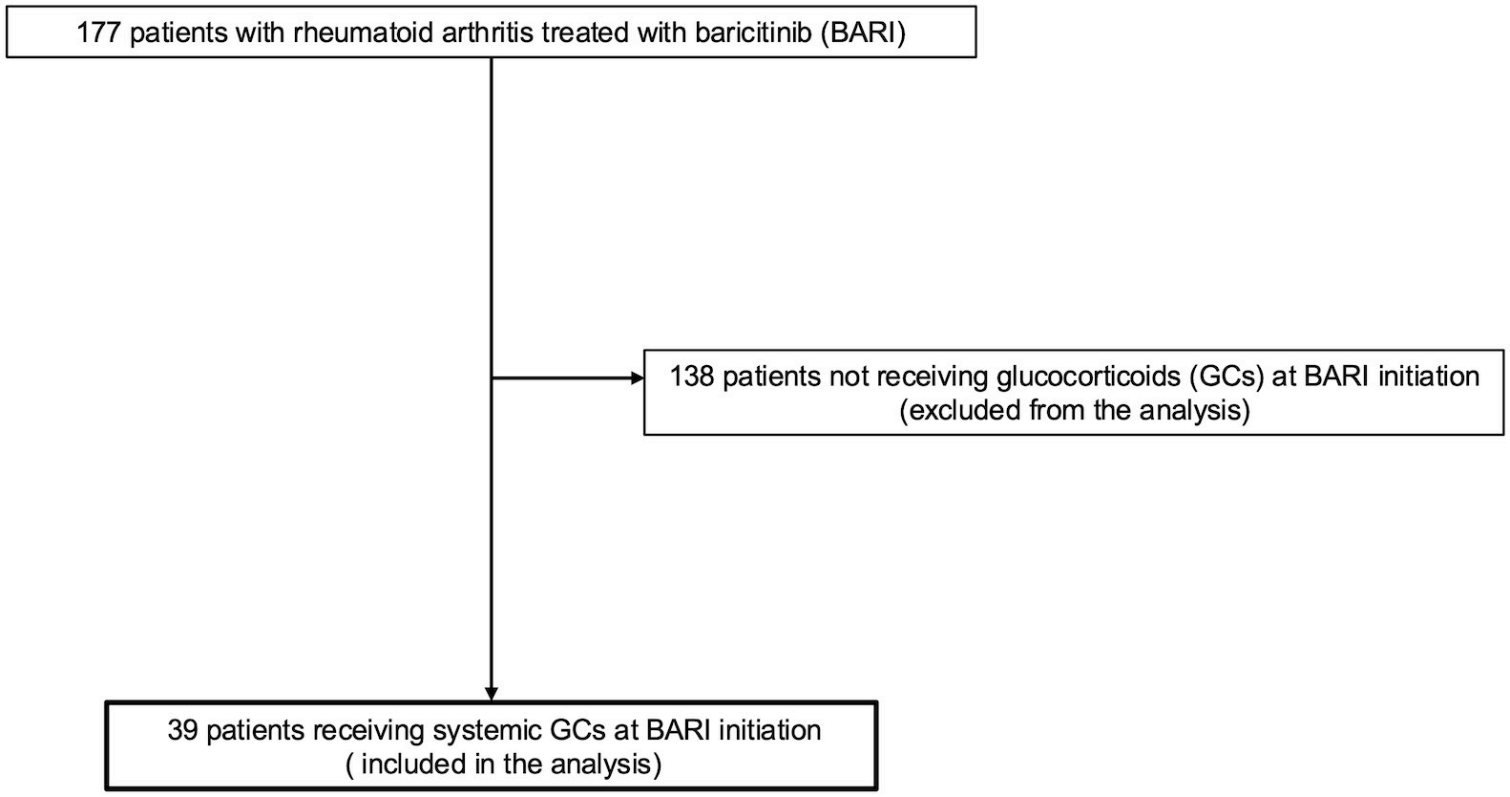
Patient flow diagram. Among 177 patients treated with BARI, 39 who were receiving glucocorticoids (GCs) at initiation were included in the analysis.

During the study period, 177 patients with RA received BARI, of whom 39 were concomitantly treated with GCs and included in this analysis (Figure 1).

The median age was 69 years, and 69% were female. The median disease duration was 7.0 years (IQR 3.5–20.5). Most patients were seropositive (87%), and the mean baseline CDAI was 20 ± 7, with 87% classified as moderate or high disease activity. The median GC dose at baseline was 3.0 mg/day (IQR 2.0–4.8). BARI was initiated at 2 mg in 23 patients (59%) and at 4 mg in 16 patients (41%). The median number of prior b/tsDMARDs was 1 (IQR 0–3). The majority of prior b/tsDMARD discontinuations were attributed to insufficient efficacy rather than adverse events, supporting the refractory characteristics of the study population. Thirty-one patients (79%) had prior exposure to at least one b/tsDMARD, and 18 (46%) were refractory to ≥ 2 agents. Comorbid ILD and CTD were present in 69% and 38% of patients, respectively (Table 1). The lower BARI dose was primarily chosen with renal impairment (*n* = 13), advanced age or the presence of comorbidities (*n* = 8), or financial reasons (*n* = 2). The CTD diagnoses included Sjögren’s disease (*n* = 5), systemic lupus erythematosus (*n* = 1), idiopathic inflammatory myositis (*n* = 5), Systemic sclerosis (*n* = 1), microscopic polyangiitis (*n* = 1), and undifferentiated CTD (*n* = 2).

**TABLE 1:** Baseline characteristics of the 39 patients treated with baricitinib plus glucocorticoids.

Variable	Value
Age, years, median (IQR)	69 (58–77)
Female, *n* (%)	27 (69%)
Disease duration, years, median (IQR)	7.0 (3.5–20.5)
Seropositive (RF and/or ACPA), *n* (%)	34 (87%)
CRP, mg/dL, median (IQR)	0.80 (0.15–2.55)
CDAI, mean ± SD	20 ± 7
CDAI LDA or remission, *n* (%)	5 (13%)
Glucocorticoid dose (prednisolone equivalent, mg/day), median (IQR)	3.0 (2.0–4.8)
Baricitinib 4 mg at initiation, *n* (%)	16 (41%)
Concomitant csDMARDs, *n* (%)	22 (56%)
Comorbid connective tissue disease, *n* (%)	15 (38%)
Comorbid interstitial lung disease, *n* (%)	27 (69%)
b/tsDMARDs-naïve, *n* (%)	8 (21%)
tsDMARDs-naïve, *n* (%)	28 (72%)
Number of prior bDMARDs, median (IQR)	1 (0–3)
Number of prior tsDMARDs, median (IQR)	0 (0–1)
Total number of prior b/tsDMARDs, median (IQR)	1 (0–3)
Refractory to ≥ 1 prior b/tsDMARDs, *n* (%)	31 (79%)
Refractory to ≥ 2 prior b/tsDMARDs, *n* (%)	18 (46%)

RF, rheumatoid factor; ACPA, anti-citrullinated protein antibody; CRP, C-reactive protein; CDAI, Clinical Disease Activity Index; LDA, low disease activity; GC, glucocorticoid; csDMARDs, conventional synthetic disease-modifying antirheumatic drugs; bDMARDs, biologic disease-modifying antirheumatic drugs; tsDMARDs, targeted synthetic disease-modifying antirheumatic drugs.

**FIGURE 2:**
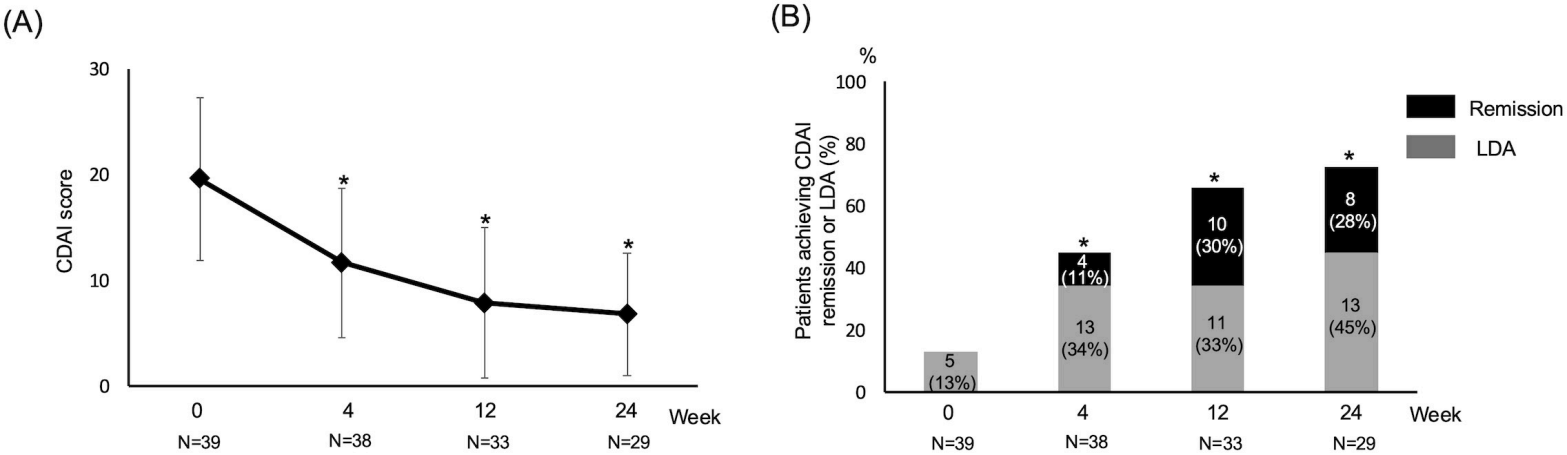
Changes in disease activity over 24 weeks after baricitinib initiation. **(A)** Changes in Clinical Disease Activity Index (CDAI) scores from baseline to week 24. **(B)** Proportions of patients achieving CDAI low disease activity (LDA) or remission over 24 weeks. Data are shown for patients with available assessments at each time point. *P < 0.05 vs. baseline.

Disease activity improved significantly after baricitinib initiation. CDAI significantly decreased from baseline to week 24 (mean 20 ± 7 to 7 ± 5, *P* < 0.001 by Friedman test), with improvement evident as early as week 4. The proportion of patients achieving CDAI remission or LDA increased from 13% at baseline to 62% at week 24 (*P* < 0.001 by Cochran’s Q test) (Figure 2).

**FIGURE 3:**
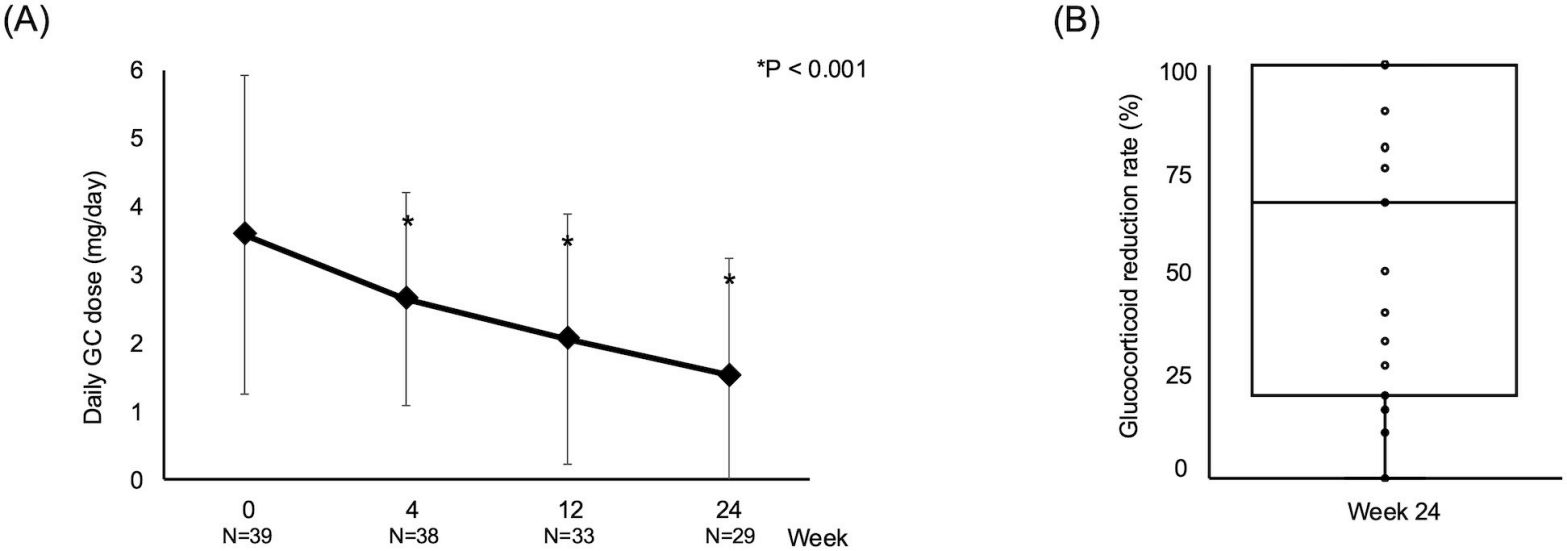
Glucocorticoid tapering with baricitinib. **(A)** Changes in daily glucocorticoid (GC) dose from baseline to week 24. **(B)** Distribution of GC reduction rates at week 24. The box represents the interquartile range with the median shown as a horizontal line. Individual data points are overlaid. *P < 0.001 vs. baseline.

The median daily GC dose decreased from 3.0 mg/day (IQR 2.0–4.8) at baseline to 1.0 mg/day (IQR 0–3.0) at week 24 (*P* < 0.001), with a significant reduction already observed at week 4 (Figure 3A). At week 24, the median GC reduction rate was 66% (interquartile range, 50%–100%). Overall, 18 patients (46%) achieved a ≥50% reduction in GC dose, and 10 (26%) discontinued GCs entirely (Figure 3B).

To identify factors associated with GC reduction at week 24, univariate linear regression analysis was performed. Refractoriness to ≥ 2 prior b/tsDMARDs was significantly associated with smaller GC reduction rates at week 24 (B = −40.26, 95% CI −65.21 to −15.31, *P* = 0.03). Other baseline factors, including ILD, CTD, seropositivity, and baseline GC dose, were not significantly associated with GC reduction. Similarly, in logistic regression analysis, baseline GC dose was not predictive of achieving GC-free status (*P* = 0.934). In two-variable multivariable models, refractoriness to ≥ 2 prior b/tsDMARDs remained independently associated with a lesser degree of GC reduction after adjustment for comorbid CTD (Model 1: B = −43.3, 95% CI −66.3 to −20.2, *P* = 0.001), comorbid ILD (Model 2: B = −40.4, 95% CI −65.9 to −14.9, *P* = 0.003), and initial BARI 4 mg use (Model 3: B = −37.9, 95% CI −64.6 to −11.3, *P* = 0.007). In contrast, the starting BARI dose (4 vs. 2 mg) was not independently associated with GC reduction after adjustment for refractoriness to ≥ 2 prior b/tsDMARD exposure (Table 2).

**TABLE 2:** Predictors of glucocorticoid reduction at week 24.

Model	Variable	B (95% CI)	*P*-value	Adjusted R^2^
Univariate	Disease duration	−0.1 (−1.0 to 0.73)	0.742	–
	Seropositive	−11.2 (−50.1 to 27.7)	0.560	–
	CDAI LDA or remission	8.7 (−20.5 to 38.0)	0.546	–
	Glucocorticoid dose	−0.9 (−6.9 to 5.1)	0.760	–
	Bari 4 mg at initiation	−19.8 (−49.0 to 9.5)	0.178	–
	b/tsDMARDs-naïve	30.4 (−2.0 to 62.8)	0.065	–
	tsDMARDs-naïve	7.6 (−26.8 to 41.9)	0.655	–
	Refractory to ≥ 1 prior b/tsDMARDs	−30.4 (−62.8 to 1.97)	0.065	–
	Refractory to ≥ 2 prior b/tsDMARDs	−40.3 (−65.2 to −15.3)	**0.003**	–
	Comorbid ILD	0.91 (−30.0 to 31.8)	0.952	–
	Comorbid CTD	−24.2 (−53.0 to 4.6)	0.096	–
Model 1	Refractory to ≥ 2 prior b/tsDMARDs	−43.3 (−66.3 to −20.2)	**0.001**	0.42
	Comorbid CTD	−29.0 (−52.7 to −5.3)	**0.018**	
Model 2	Refractory to ≥ 2 prior b/tsDMARDs	−40.4 (−65.9 to −14.9)	**0.003**	0.28
	Comorbid ILD	2.9 (−23.8 to 29.7)	0.824	
Model 3	Refractory to ≥ 2 prior b/tsDMARDs	−37.9 (−64.6 to −11.3)	**0.007**	0.29
	Bari 4 mg at initiation	−7.6 (−34.5 to 19.8)	0.575	

Results of univariate and two-variable linear regression analyses. Values are regression coefficients (B) with 95% confidence intervals (CI). Models 1–3 include “refractoriness to ≥ 2 prior b/tsDMARDs” and one additional variable (CTD, ILD, or BARI dose). Bold values indicate statistical significance (*P* < 0.05). BARI, baricitinib; b/tsDMARDs, biologic or targeted synthetic disease-modifying antirheumatic drugs; ILD, interstitial lung disease; CTD, connective tissue disease.

During follow-up, 10 patients discontinued BARI: 6 due to insufficient efficacy, 3 due to adverse events (elevated liver enzymes, increased creatine kinase, and worsening ILD, one each), and 1 for financial reasons. No unexpected safety signals were observed.

## Discussion

Previous studies have shown that among bDMARDs, interleukin-6 inhibitors (IL-6i) have the greatest GC-sparing potential, but marked reduction in GC dose is achieved only in a subset of patients (17). A recent comparative study demonstrated that over 50% of patients treated with JAK inhibitors discontinued GCs within one year, compared with approximately 40% of those treated with bDMARDs (18).

In contrast to these reports, the present study focused on a population with clinical features predictive of difficulty in GC tapering, including a high rate of seropositivity (84%), long disease duration (median 7 years), and frequent comorbidities such as ILD (69%) and CTD (38%). Excessive GC tapering, particularly to doses ≤ 2.5 mg/day, has been associated with an increased risk of disease flare in patients receiving biologic DMARDs (19), and accordingly, large Japanese RA cohorts have reported that approximately half of patients remain on GC therapy two years after bDMARD initiation (20). Consistent with these observations, despite prior exposure to b/tsDMARDs in 79% of patients (including ≥ 2 agents in 46%), 87% still had moderate or higher disease activity by CDAI and could not reduce the median GC dose below 3 mg/day at BARI initiation. In contrast, recent studies of the GC-sparing effects of BARI and other JAK inhibitors included cohorts in which approximately 50% of patients were receiving concomitant GCs at treatment initiation (15, 17, 20), whereas only 22% of patients in our cohort were on GCs at initiation. These findings suggest that our cohort represents a particularly refractory population in whom GC discontinuation could not be achieved despite aggressive use of prior therapies.

Under these circumstances, we evaluated GC reduction rates over a relatively early 24-week period. The median GC reduction rate was 66%, and 33% of patients achieved GC discontinuation. Although the discontinuation rate was slightly lower than that reported in previous studies, this finding remains clinically meaningful given the difficult-to-treat characteristics of the cohort. Notably, baseline GC dose was not associated with the likelihood of achieving complete discontinuation. This suggests that baseline dose alone may not be a major barrier to GC tapering when adequate disease control is achieved with BARI. Given the relatively low baseline GC doses in our cohort (median 3 mg/day), the ability to detect dose-dependent effects may have been limited. Furthermore, CDAI scores improved significantly, with 62% of patients achieving remission or low disease activity, confirming that GC tapering was accompanied by effective disease control.

With respect to factors associated with GC reduction, refractoriness to ≥ 2 b/tsDMARDs was independently associated with a lower degree of GC reduction, consistent with the definition and clinical profile of D2T-RA (10). These findings underscore the importance of introducing JAK inhibitors before the accumulation of multiple treatment failures to achieve successful GC tapering and potentially prevent progression to a D2T state. This interpretation is supported by recent registry data demonstrating that JAK inhibitors remain effective after inadequate response to first-line b/tsDMARDs, and that switching between JAK inhibitors yields better outcomes than switching to another bDMARD (21–24).

In contrast, comorbid ILD did not significantly affect the GC reduction rate in our analysis. This finding is consistent with previous reports demonstrating the safety and efficacy of JAK inhibitors, including BARI, in patients with RA-ILD (25, 26). In addition, emerging evidence suggests that JAK2 signaling contributes to the pathogenesis of fibrotic lung disease, particularly in RA-ILD, supporting a potential anti-fibrotic role of JAK2 blockade (27).

By comparison, comorbid CTD was associated with a smaller degree of GC reduction. Anti-nucleolar antibody (ANA) positivity has been associated with inadequate response to bDMARDs (28). Although some studies have suggested that tsDMARDs may be effective in ANA-positive or CTD-associated RA through modulation of interferon-related pathways (29), in the present study the CTD group was heterogeneous, and its association with GC tapering should be interpreted with caution.

In our cohort, the median age was 69 years, and a substantial proportion of patients had impaired renal function, which resulted in frequent use of BARI monotherapy and the 2 mg dosing. Similar patient characteristics and dosing patterns have been reported in recent real-world studies, in which 30–40% of patients received BARI as monotherapy and achieved favorable clinical outcomes (22, 23, 30).

In patients with renal impairment, who represent the primary indication for dose reduction to 2 mg, recent pharmacokinetic and real-world data have demonstrated comparable efficacy and treatment persistence between the 2 and 4 mg doses of BARI (31). Although dose-stratified analysis was not feasible in the present study due to the limited number of GC-dependent cases, the aim of this study was not to compare dosing regimens but to evaluate the GC-sparing effect of BARI under routine clinical conditions. In this context, the achievement of meaningful GC reduction despite heterogeneous dosing supports the clinical relevance of BARI in real-world practice.

In terms of safety, our findings are consistent with prior long-term studies showing a stable safety profile for BARI over a median 4.6 years of exposure (32). Recent registry-based analyses have reported comparable rates of adverse events and treatment discontinuation between JAK inhibitors and bDMARDs (24, 33). However, large randomized safety trials have suggested an increased risk of major adverse cardiovascular events in certain high-risk populations treated with JAK inhibitors, underscoring the importance of careful patient selection and risk stratification (34). Consistent with these data, no unexpected safety signals were observed in our cohort.

This study has several limitations. First, it was an analysis of a small number of cases from a single institution. However, given current RA treatment strategies that aim to minimize GC use, it is inherently difficult to construct large cohorts of patients who remain GC-dependent in daily clinical practice. Second, GC tapering protocols were not standardized across patients, reflecting real-world clinical decision-making. While this heterogeneity limits strict comparisons, it also enhances the generalizability of our findings to daily practice. Moreover, as this was not a blinded controlled study, expectations from physicians and patients regarding GC reduction may have influenced tapering decisions and contributed to the observed reductions. Third, analyses were conducted using an on-treatment approach without imputation for missing data. Because six patients discontinued due to insufficient efficacy, GC reduction may have been slightly overestimated, although the discontinuation rate (15%) was consistent with real-world data. Finally, our analysis focused primarily on refractoriness to prior b/tsDMARDs as a determinant of D2T-RA. The EULAR concept of D2T-RA encompasses broader dimensions beyond treatment refractoriness, including factors such as fatigue and comorbidity burden, which have been highlighted in an international EULAR survey but were not fully captured in the present study (35). Future studies incorporating these multidimensional clinical factors may further clarify the comprehensive role of baricitinib in the management of D2T-RA.

In this real-world cohort of RA patients who remained GC-dependent despite prior therapies, BARI achieved clinically meaningful GC reduction within 24 weeks and was associated with significant improvements in disease activity. Refractoriness to two or more prior b/tsDMARDs was identified as a key barrier to GC tapering, highlighting the importance of introducing JAK inhibitors before progression to a D2T state. BARI was well tolerated, and no unexpected safety signals were observed.

## Data Availability

The raw data supporting the conclusions of this article will be made available by the authors, without undue reservation.
